# DANE-MDA: Predicting microRNA-disease associations via deep attributed network embedding

**DOI:** 10.1016/j.isci.2021.102455

**Published:** 2021-04-20

**Authors:** Bo-Ya Ji, Zhu-Hong You, Yi Wang, Zheng-Wei Li, Leon Wong

**Affiliations:** 1 Xinjiang Technical Institutes of Physics and Chemistry, Chinese Academy of Sciences, Urumqi 830011, China; 2 University of the Chinese Academy of Sciences, Beijing 100049, China; 3 Xinjiang Laboratory of Minority Speech and Language Information Processing, Urumqi 830011, China; 4 School of Computer Science and Technology, China University of Mining and Technology, Xuzhou 221116, China

**Keywords:** Computational bioinformatics, Systems biology, Cancer

## Abstract

Predicting the microRNA-disease associations by using computational methods is conductive to the efficiency of costly and laborious traditional bio-experiments. In this study, we propose a computational machine learning-based method (DANE-MDA) that preserves integrated structure and attribute features via deep attributed network embedding to predict potential miRNA-disease associations. Specifically, the integrated features are extracted by using deep stacked auto-encoder on the diverse orders of matrixes containing structure and attribute information and are then trained by using random forest classifier. Under 5-fold cross-validation experiments, DANE-MDA yielded average accuracy, sensitivity, and AUC at 85.59%, 84.23%, and 0.9264 in term of HMDD v3.0 dataset, and 83.21%, 80.39%, and 0.9113 in term of HMDD v2.0 dataset, respectively. Additionally, case studies on breast, colon, and lung neoplasms related disease show that 47, 47, and 46 of the top 50 miRNAs can be predicted and retrieved in the other database.

## Highlights

• A computational machine learning-based method for miRNA-disease association prediction
• Preserve structure and attribute features via deep attributed network embedding
• Capture the interaction between two kinds of features from diverse degrees of proximity
• Extract the higher-order features via deep stacked auto-encoder neural network

## Graphical abstract

**Figure:**
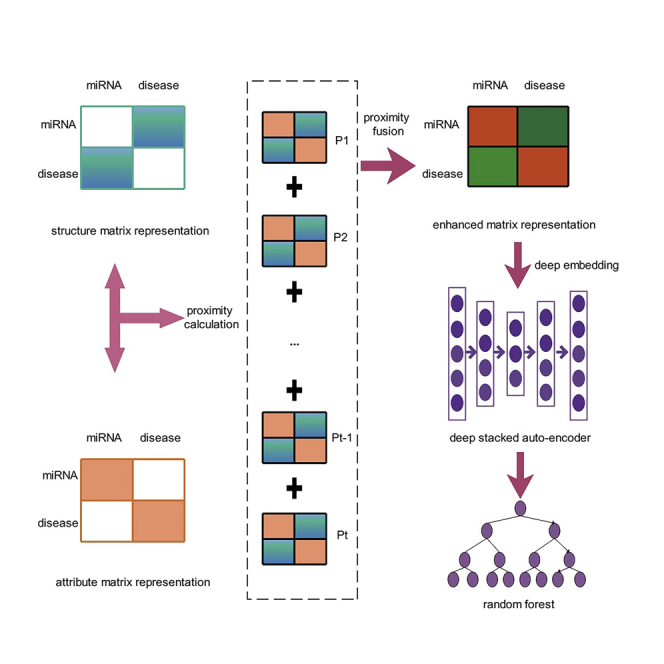

## Introduction

The human genomes have various endogenous “non-messenger” or “non-coding” RNAs, including a large number of single-stranded microRNAs (miRNAs) containing about 22 nucleotides (Ambros, 2001, 2004). miRNAs play a significant function in various human life processes, including virus defense, tissue development, cell metabolism, and organ formation, and participate in the regulation of post-transcriptional gene expression (Cui et al., 2006; Karp and Ambros, 2005; Lu et al., 2005; Rupaimoole and Slack, 2017; Xu et al., 2004). Furthermore, miRNAs also have a particular therapeutic impact as a regulator for several genes (Ling et al., 2013; Matsui and Corey, 2017). A cascade of studies have shown that miRNAs can become drug targets for human disease treatments (Mishra et al., 2020), hence it is not surprising that predicting and identifying potential miRNAs related to corresponding diseases have been the focus of researchers. For example, Jeong et al. (Jeong et al., 2011) stated that let-7a is under-expressed in the tissues and cells of patients with NSCLC (non-small cell lung cancer) compared with the normal control group. Bang et al. (2012) found that the miR-23/27/24 cluster is related to retinal vascular development and endothelial cell apoptosis and angiogenesis in cardiac ischemia. In recent years, massive miRNA-disease associations have been acquired through traditional biological experiments and stored in public databases. These biological experimental methods usually have high prediction accuracy; nevertheless, their processes are complex, expensive, and time-consuming (Liang et al., 2019). To this end, to accelerate the verification process, and reduce the time consumption and blindness of biological experiments, it is significant to establish computational methods for quickly and effectively predicting possible miRNA-disease associations (Wong et al., 2020; Yi et al., 2020).

Taking advantage of the hypothesis that functionally related miRNAs are more likely to be related to diseases with similar phenotypes, some score function-based computational models have been proposed for predicting miRNA-disease associations, which commonly leverage methods such as random walk to calculate the likelihood of potential associations on the constructed miRNA-disease association network. For example, Chen et al. (2012) first incorporated known miRNA-disease associations and large-scale miRNA-miRNA functional similarity information and then utilized the random walk and global network similarity measure methods to obtain superior performance than previous models. Luo et al. (2017) assessed the similarity between diseases or miRNAs by incorporating several relevant heterogeneous information. Then, a semi-supervised mechanics of Kronecker regularized least squares was employed to predict possible miRNAs related to diseases. Wang et al. (2019) utilized the logical trees classifier and fused the known miRNA-disease association, miRNA functional similarity and sequence information, and disease semantic similarity to predict miRNA-disease associations. Empirical results of cross-validation experiments and case studies both demonstrated the reliability and effectiveness of their model. Alaimo et al. (2014) adopted a recommendation algorithm to predict novel associations between miRNAs and diseases based on a tripartite network composed of miRNAs, targets, and diseases, where the targets act as intermediate nodes between miRNAs and diseases. On this basis, a multi-level resource transfer method was employed to compute the correlation degree between each miRNA-disease pair.

Recently, machine learning and deep learning also have been utilized for predicting possible associations between miRNAs and diseases with the growth of known miRNA-disease association data. For example, Xu et al. (2011) calculated four topological features of miRNAs and then trained the gold-standard miRNA dataset using the support vector machine (SVM) for predicting possible miRNA-disease associations. To break the restriction of previous models that cannot be applied for diseases without any known associated miRNAs, Chen and Yan (2014) exploited the least-squares regularization and semi-supervised learning method to reveal the miRNA-disease associations and obtain reliable performance. These existing models almost utilized miRNA functional similarity, miRNA-family associations, disease semantic similarity, miRNA-target associations, and known miRNA-disease associations. However, the known miRNA-disease associations are not well mined. These known miRNA-disease associations can be constructed as a graph or network, but the node features in the graph are rarely calculated. Therefore, some of the recent techniques in graph embedding are used for predicting miRNA-disease associations, such as graph convolutional networks (Kipf and Welling, 2016), matrix factorization (He et al., 2018, 2019), and Bayesian learning (Hu et al., 2019). For example, Xuan et al. (2019) utilized convolutional neural networks and network representation learning to design a computational model to predict miRNA-disease associations. Zheng et al. (2020a) exploited the graph embedding method and random forest classifier to reveal novel miRNA and disease associations. Their method gained good performance by combining the behavior and attribute features of diseases and miRNAs.

**Figure 1:**
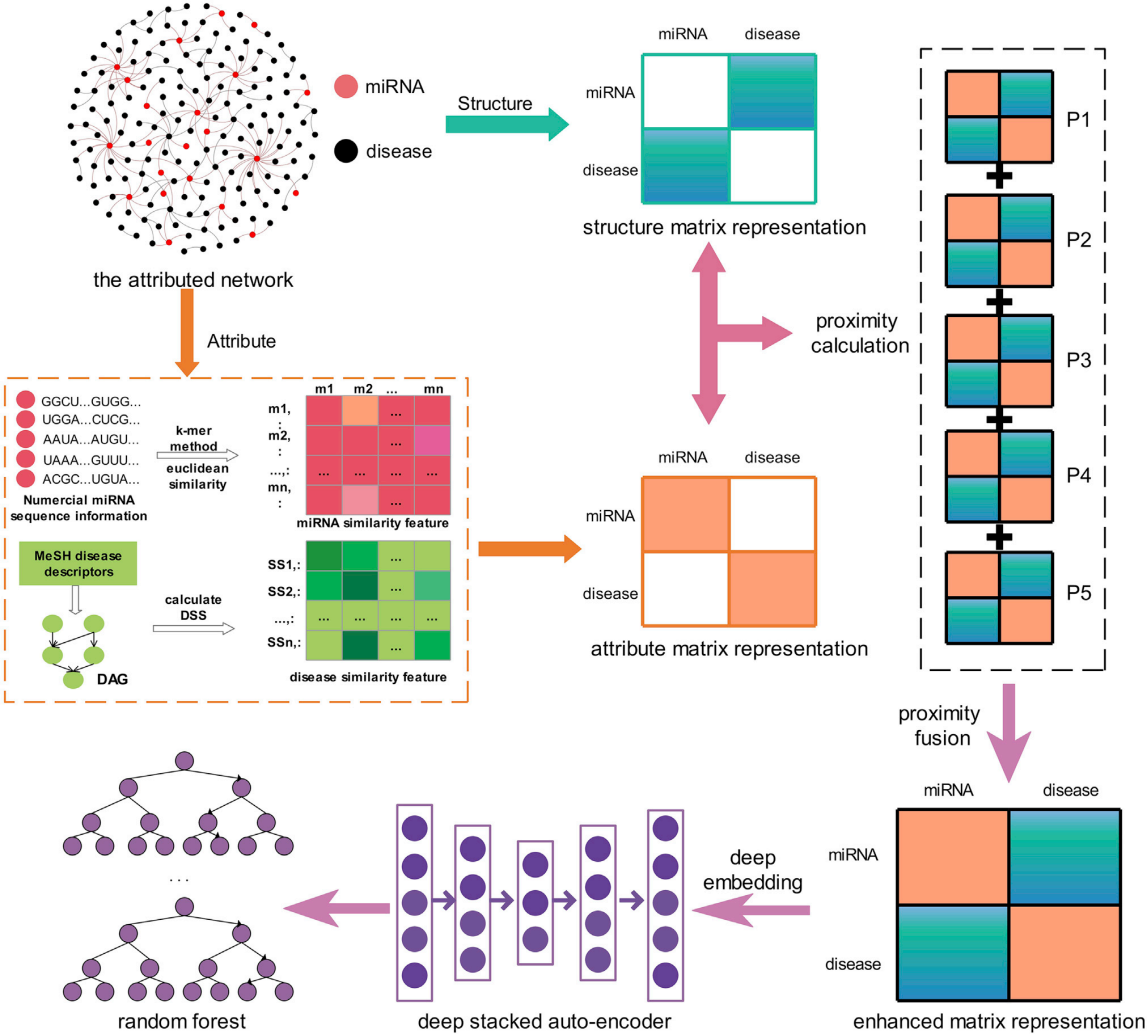
Illustration of the overall framework of DANE-MDA (DAG: directed acyclic graph; DSS: disease semantic similarity)

In this study, we propose a computational machine learning-based method (DANE-MDA) that attempts to preserve both the diverse degrees of network structure and attribute feature of miRNAs and diseases via deep attributed network embedding to predict potential miRNA-disease associations. DANE-MDA includes four steps. First, we constructed an attributed network by connecting the known miRNA-disease associations in the Human MicroRNA Disease Database (HMDD) and, respectively, calculated the attribute and network structure feature of miRNAs and diseases, where the attribute feature includes miRNA sequence similarity and disease semantic similarity and the network structure feature includes the probability of direct transition between each miRNA-disease association pair. Second, we captured the interactions between network structure and attribute information of miRNAs and diseases from diverse degrees of proximity by utilizing a personalized random walk-based method. Third, we fused the various degrees of proximity to build an enhanced matrix representation, which contains both the attribute feature, as well as the local and global network structure feature of miRNAs and diseases and then exploited the deep stacked auto-encoder to learn the complex and nonlinear information in the enhanced matrix to represent miRNAs and diseases. Finally, the Random Forest classifier is selected to construct the prediction model. The illustration of the DANE-MDA overall framework is shown in Figure 1. As a result, the 5-fold cross-validation experiment was applied to examine the performance of DANE-MDA, which obtained an average 85.59% accuracy, 84.23% sensitivity, and 0.9264 area under the receiver operating characteristic (ROC) curve (AUC) on the HMDD v3.0 dataset, and an average 83.21% accuracy, 80.39% sensitivity, and 0.9113 AUC on the HMDD v2.0 dataset. What's more, we also conducted case studies on three common human diseases, including breast, colon, and lung neoplasms, to verify the performance of DANE-MDA in practical applications. Additionally, we also compared the influence of model parameters and classifiers on prediction results. In summary, the proposed DANE-MDA model has a promising performance for predicting novel miRNA-disease associations and is anticipated to be an effective supplement tool in the field of bioinformatics research.

## Results

### The results of DANE-MDA under 5-fold cross-validation experiment

**Figure 2:**
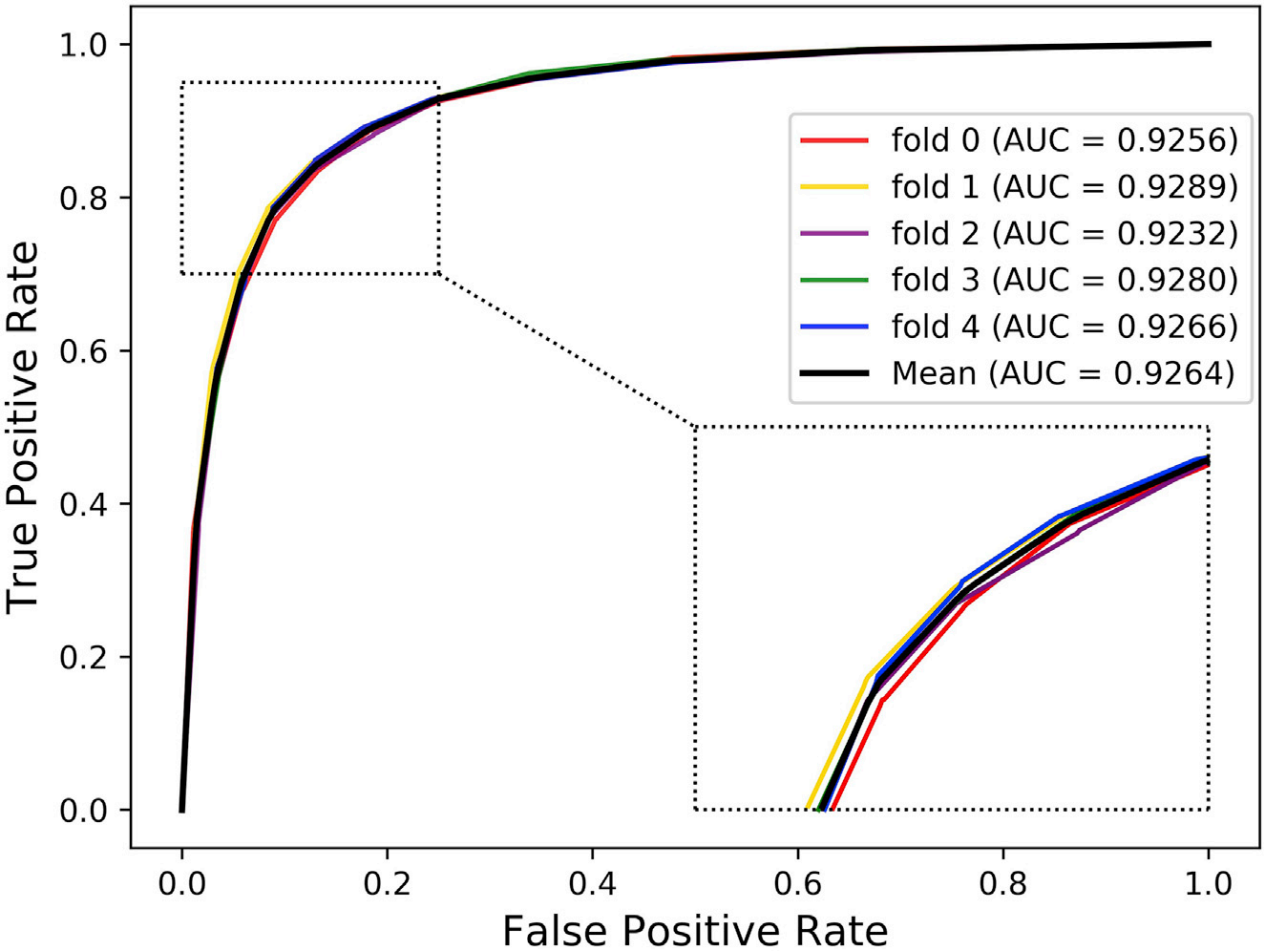
The ROC curves of DANE-MDA under 5-fold cross validation based on HMDD v3.0 dataset

**Figure 3:**
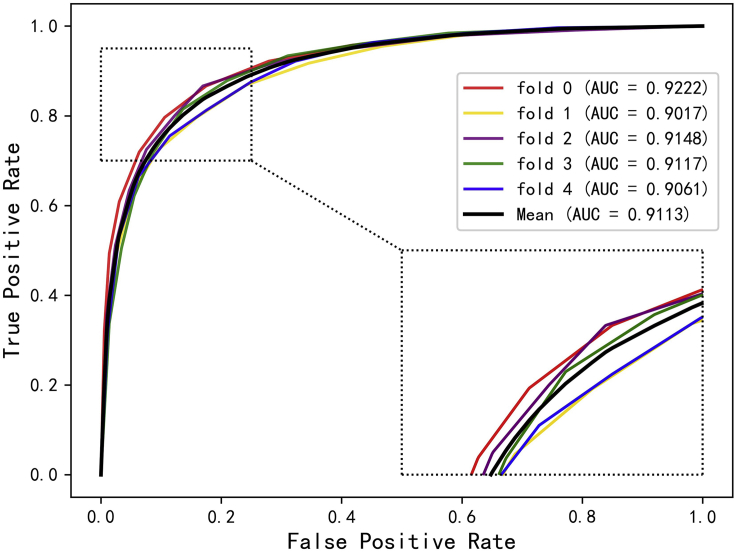
The ROC curves of DANE-MDA under 5-fold cross validation based on HMDD v2.0 dataset

**Figure 4:**
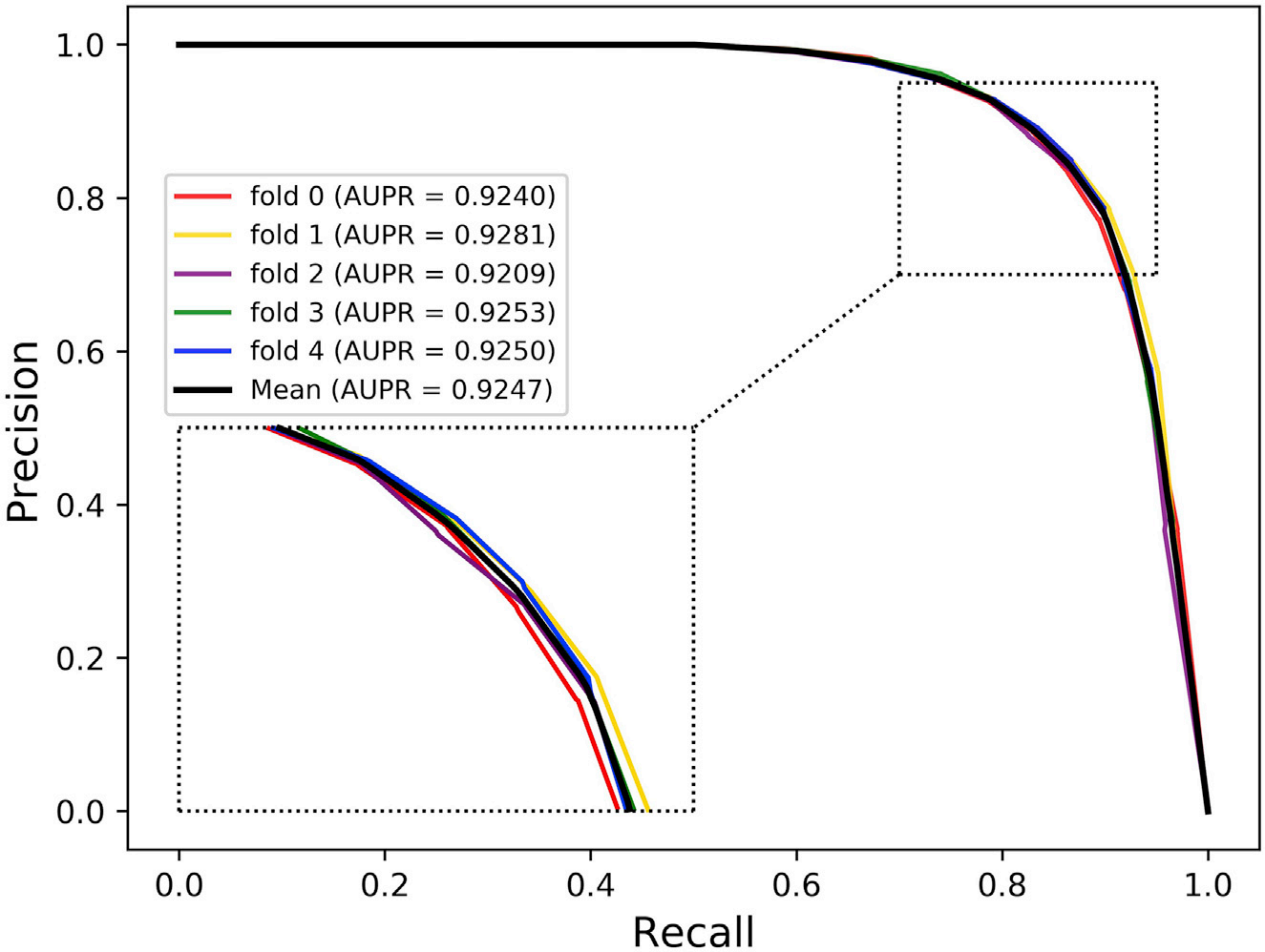
The PR curves of DANE-MDA under 5-fold cross validation based on HMDD v3.0 dataset

**Figure 5:**
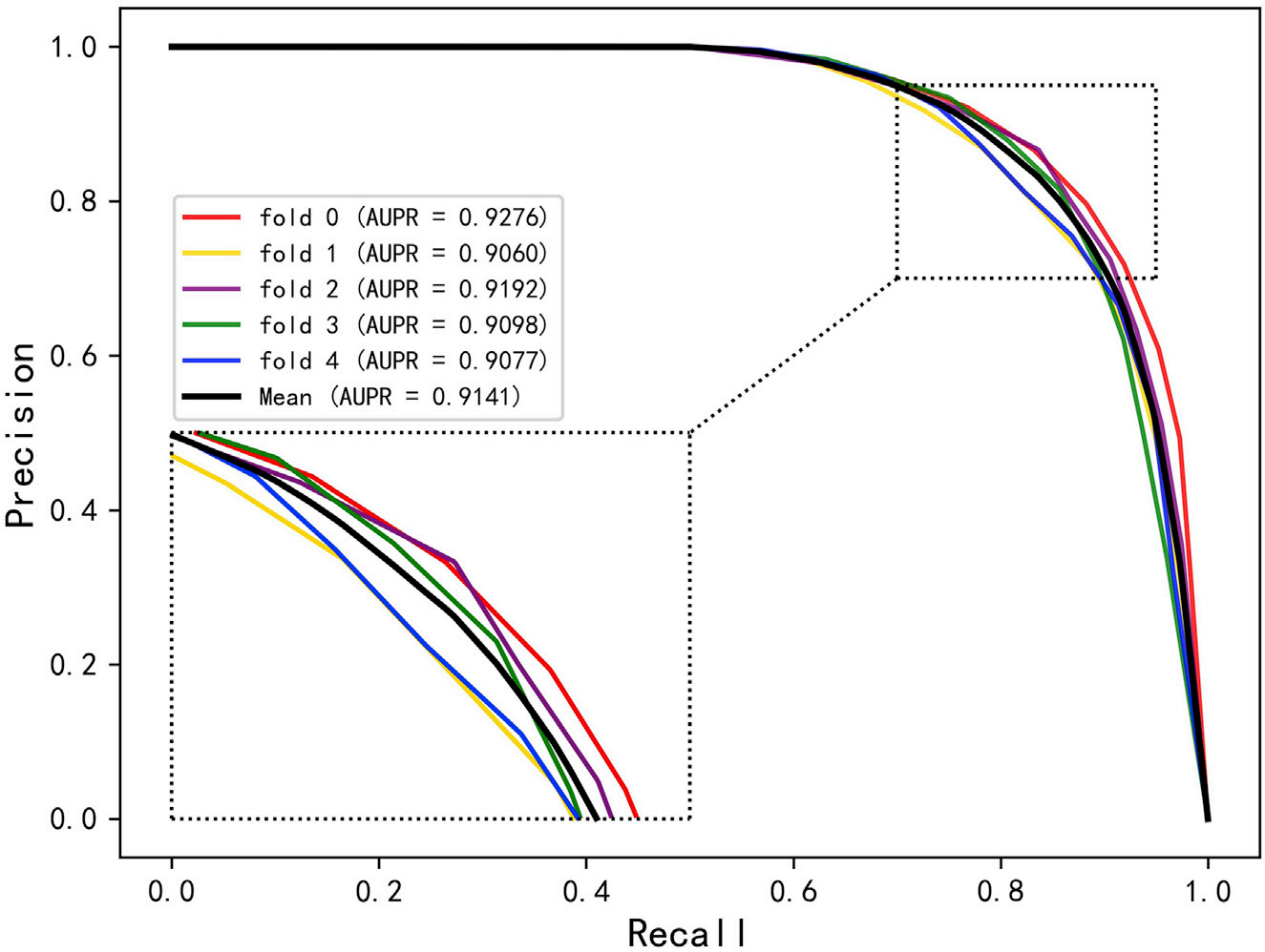
The PR curves of DANE-MDA under 5-fold cross validation based on HMDD v2.0 dataset

Cross-validation is a common method for building models and verifying model parameters in machine learning (Cooil et al., 1987). In this study, the 5-fold cross-validation experiment is implemented to evaluate the ability of DANE-MDA for predicting novel miRNA-disease associations. Specifically, the positive and negative samples are, respectively, separated into five folds, one fold is the test dataset and the rest four folds are the training dataset. On this basis, five experiments are respectively performed in sequence. In the results, six evaluation indicators in each fold experiment including Accuracy (Acc.), Precision (Prec.), Matthews Correlation Coefficient (MCC), Specificity (Spec.), Sensitivity (Sen.), and the AUC based on the HMDD v3.0 and v2.0 dataset are, respectively, recorded in Tables 1 and 2. Furthermore, the ROC and precision-recall (PR) curve is further selected to verify the prediction ability of DANE-MDA. Figures 2, 3, 4, and 5 respectively show the 5-fold cross-validation ROC and PR curves of DANE-MDA based on the HMDD v3.0 and v2.0, which, respectively, draws the sensitivity (true positive rate) against the specificity (false positive rate) and the precision against the recall under various score thresholds.

**Table 1:** The results of DANE-MDA under 5-fold cross-validation based on the HMDD v3.0 dataset

Fold	ACC.(%)	AUC(%)	Sen.(%)	Prec.(%)	Spec.(%)	MCC(%)
0	85.10	92.56	83.32	86.40	86.88	70.25
1	85.94	92.89	84.57	86.95	87.31	71.91
2	85.38	92.32	83.48	86.78	87.28	70.81
3	85.59	92.80	84.88	86.11	86.31	71.19
4	85.96	92.66	84.89	86.74	87.02	71.93
**Average**	**85.59 ± 0.37**	**92.64 ± 0.22**	**84.23 ± 0.77**	**86.60 ± 0.34**	**86.96 ± 0.41**	**71.22 ± 0.72**

The last line represents the average and standard deviation of each indicator.

**Table 2:** The results of DANE-MDA under 5-fold cross-validation based on the HMDD v2.0 dataset

Fold	ACC.(%)	AUC(%)	Sen.(%)	Prec.(%)	Spec.(%)	MCC(%)
0	84.53	92.22	79.65	88.27	89.41	69.39
1	81.86	90.17	79.56	83.40	84.16	63.79
2	83.89	91.48	80.02	86.73	87.75	67.98
3	83.93	91.17	81.49	85.67	86.37	67.94
4	81.86	90.61	81.22	82.28	82.50	63.73
**Average**	**83.21 ± 1.26**	**91.13 ± 0.79**	**80.39 ± 0.90**	**85.27 ± 2.44**	**86.04 ± 2.76**	**66.57 ± 2.63**

The last line represents the average and standard deviation of each indicator.

### The impact of model parameters on prediction results

**Figure 6:**
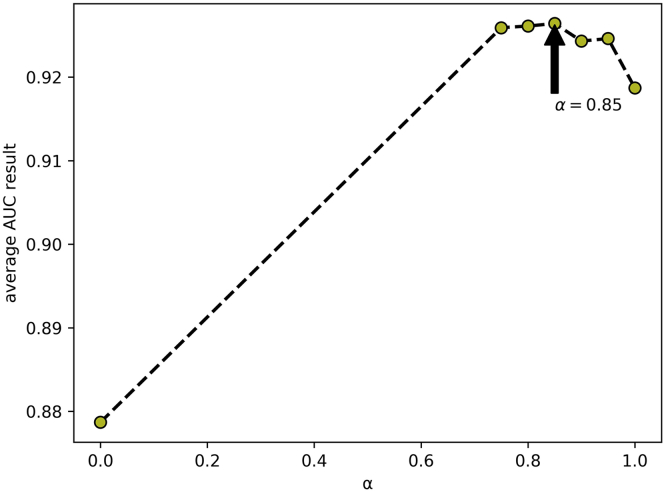
The line graph of average AUC results at different α values of DANE-MDA

**Figure 7:**
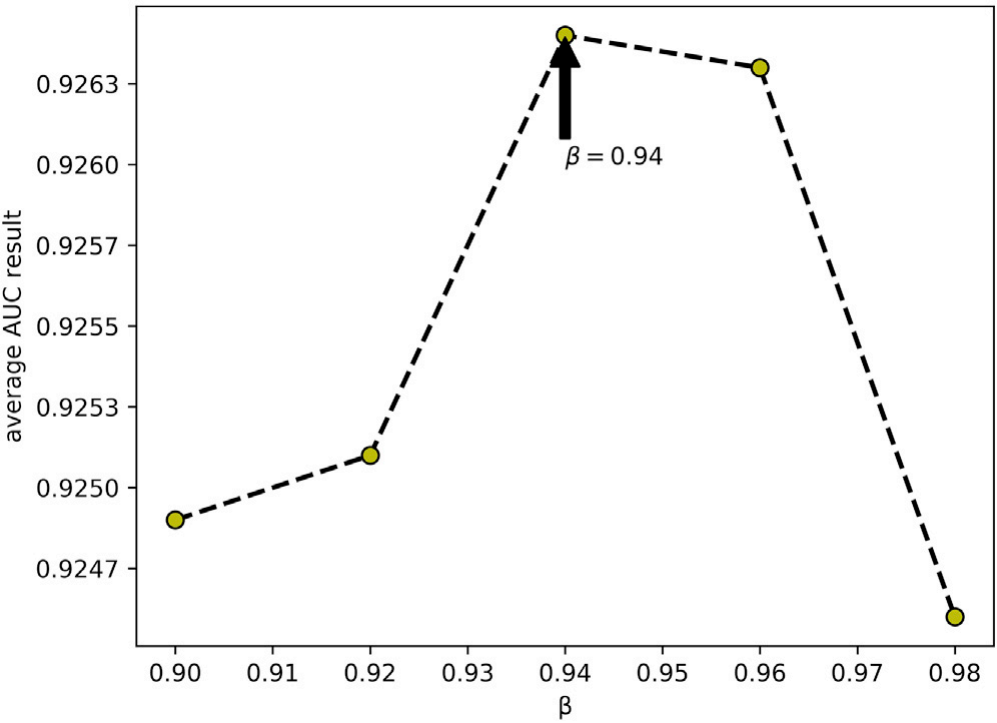
The line graph of average AUC results at different β values of DANE-MDA

**Figure 8:**
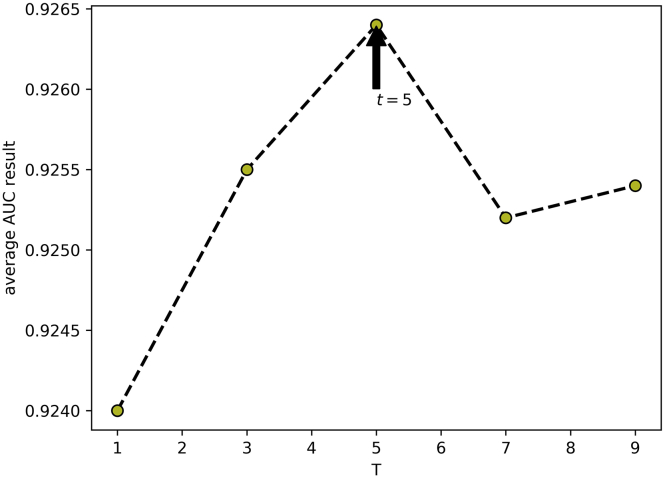
The line graph of average AUC results at different t values of DANE-MDA

In this part, we quantitatively analyzed the influence of the parameters in DANE-MDA on the prediction performance, including *α, β*, and *t*. Respectively, to fuse the network structure feature and attribute information of miRNAs and diseases, we introduced the weight parameter *α* to represent the preference ratio between attribute and structural information, with a value between 0 and 1. When *α* = 1, the predictive ability of DANE-MDA entirely depends on the structure information, and when *α* = 0, the predictive ability of DANE-MDA entirely depends on the attribute information. Moreover, the parameter *t* is introduced to capture global network structure information. Intuitively, the larger the value of *t*, the more global structure information will be obtained. However, when *t* gradually increases, the global information obtained gradually becomes weaker, and excess noise information will cause the prediction results to decrease. Last, because the low-order network structure feature is more influential than the high-order ones, we introduced the parameter *β* to control the downtrend of higher-order information, with a value between 0 and 1. On this basis, we, respectively, selected the following parameters to perform 5-fold cross-validation:*α*∈{1, 0.95, 0.90, 0.85, 0.80, 0.75, 0}, *β*∈{0.98, 0.96, 0.94, 0.92, 0.90},*t*∈{1, 3, 5, 7, 9} and used the AUC value as the evaluation indicator. For each parameter, other parameters and the experimental environment are controlled to be consistent. Tables 3, 4, and 5, respectively, show the distribution of the AUC values for each cross-validation. Additionally, the line curve of the mean AUC value was shown in Figures 6, 7, and 8. In the results, for parameter *α*, when *α* = 0.85 (fusion of 85% network structure and 15% attribute feature), DANE-MDA obtains the best performance. For parameter *β*, when *β* = 0.94, DANE-MDA has the best control over the downward trend of high-order features. For parameter *t*, when *t* = 5, DANE-MDA obtains the optimal global structural features.

**Table 3:** The AUC values of parameter *α* under each fold cross-validation (*β* = 0.94, *t* = 5)

Fold ***α***	0	1	2	3	4	Average
1	0.9169	0.9224	0.9149	0.9223	0.9171	0.9187 ± 0.34
0.95	0.9242	0.9263	0.9206	0.9269	0.9252	0.9246 ± 0.25
0.90	0.9211	0.9272	0.9230	0.9286	0.9215	0.9243 ± 0.34
**0.85**	**0.9256**	**0.9289**	**0.9232**	**0.9280**	**0.9266**	**0.9264 ± 0.22**
0.80	0.9271	0.9277	0.9243	0.9270	0.9241	0.9261 ± 0.17
0.75	0.9262	0.9299	0.9224	0.9250	0.9261	0.9259 ± 0.27
0	0.8774	0.8849	0.8776	0.8791	0.8746	0.8787 ± 0.38

**Table 4:** The AUC values of parameter *β* under each fold cross-validation (*α* = 0.85, *t* = 5)

Fold **β**	0	1	2	3	4	Average
0.98	0.9274	0.9253	0.9208	0.9275	0.9222	0.9246 ± 0.30
0.96	0.9249	0.9312	0.9252	0.9279	0.9222	0.9263 ± 0.34
**0.94**	**0.9256**	**0.9289**	**0.9232**	**0.9280**	**0.9266**	**0.9264 ± 0.22**
0.92	0.9249	0.9252	0.9221	0.9291	0.9243	0.9251 ± 0.25
0.90	0.9234	0.9268	0.9238	0.9279	0.9224	0.9249 ± 0.24

**Table 5:** The AUC values of parameter *t* under each fold cross-validation ()

Fold **t**	0	1	2	3	4	Average
1	0.9247	0.9260	0.9210	0.9290	0.9193	0.9240 ± 0.39
3	0.9255	0.9286	0.9236	0.9250	0.9249	0.9255 ± 0.19
**5**	**0.9256**	**0.9289**	**0.9232**	**0.9280**	**0.9266**	**0.9264 ± 0.22**
7	0.9234	0.9282	0.9213	0.9307	0.9223	0.9252 ± 0.41
9	0.9264	0.9277	0.9202	0.9292	0.9234	0.9254 ± 0.36

**Figure 9:**
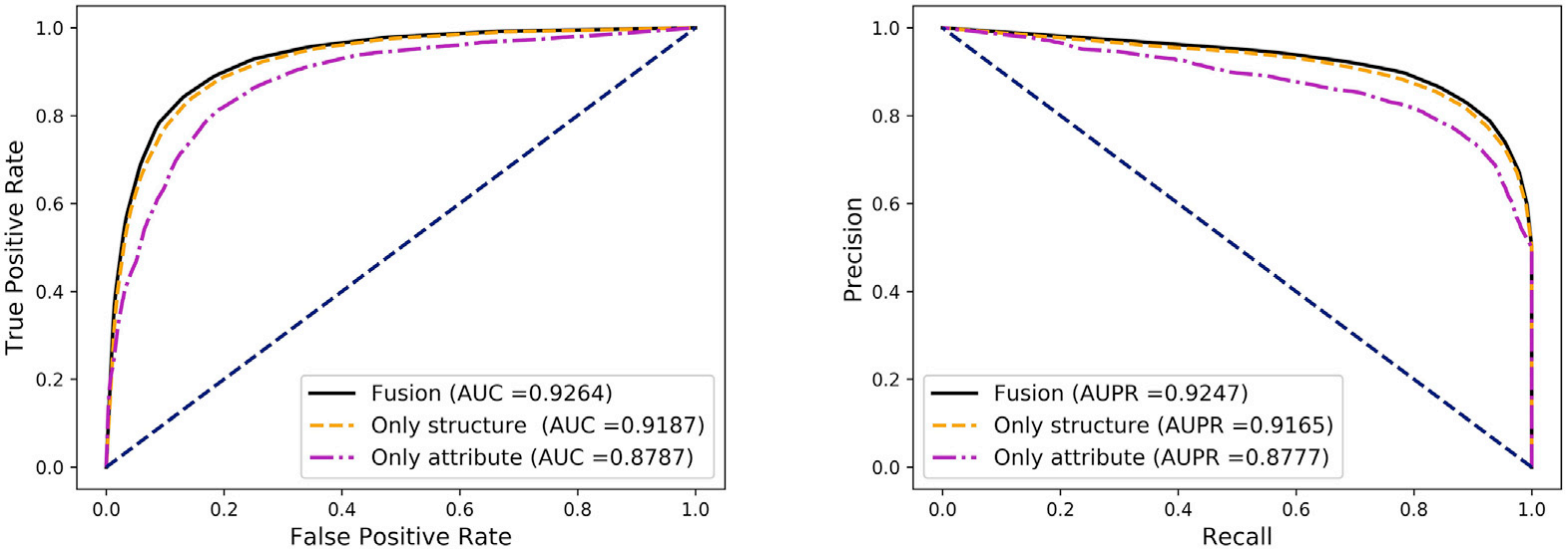
The average ROC and PR curves of DANE-MDA with different feature combinations under 5-fold cross-validation

Furthermore, to further describe the effectiveness of our feature fusion strategy, we displayed the performance of DANE-MDA with three different feature combinations under the 5-fold cross-validation: only attribute features of miRNAs and diseases (*α* = 0), only network structure features of miRNAs and diseases (*α* = 1), and the fusion feature of attribute and structure information (*α* = 0.85). The detailed average prediction results were shown in Table 6. Additionally, Figure 9 showed the ROC and PR curves of the comparative experiment. The empirical results further proved the better performance of our feature fusion strategy.

**Table 6:** The average results and standard deviations of DANE-MDA with different feature combinations under 5-fold cross-validation

Feature	Acc.(%)	AUC(%)	Sen.(%)	Prec.(%)	Spec.(%)	MCC(%)
Only attribute	81.01 ± 0.28	87.87 ± 0.38	81.86 ± 0.91	80.49 ± 0.37	80.15 ± 0.63	62.03 ± 0.58
Only structure	84.76 ± 0.21	91.87 ± 0.34	83.39 ± 0.39	85.75 ± 0.31	86.14 ± 0.38	69.55 ± 0.42
**Fusion**	**85.59 ± 0.37**	**92.64 ± 0.22**	**84.23 ± 0.77**	**86.60 ± 0.34**	**86.96 ± 0.41**	**71.22 ± 0.72**

### The impact of the classifier on prediction results

**Figure 10:**
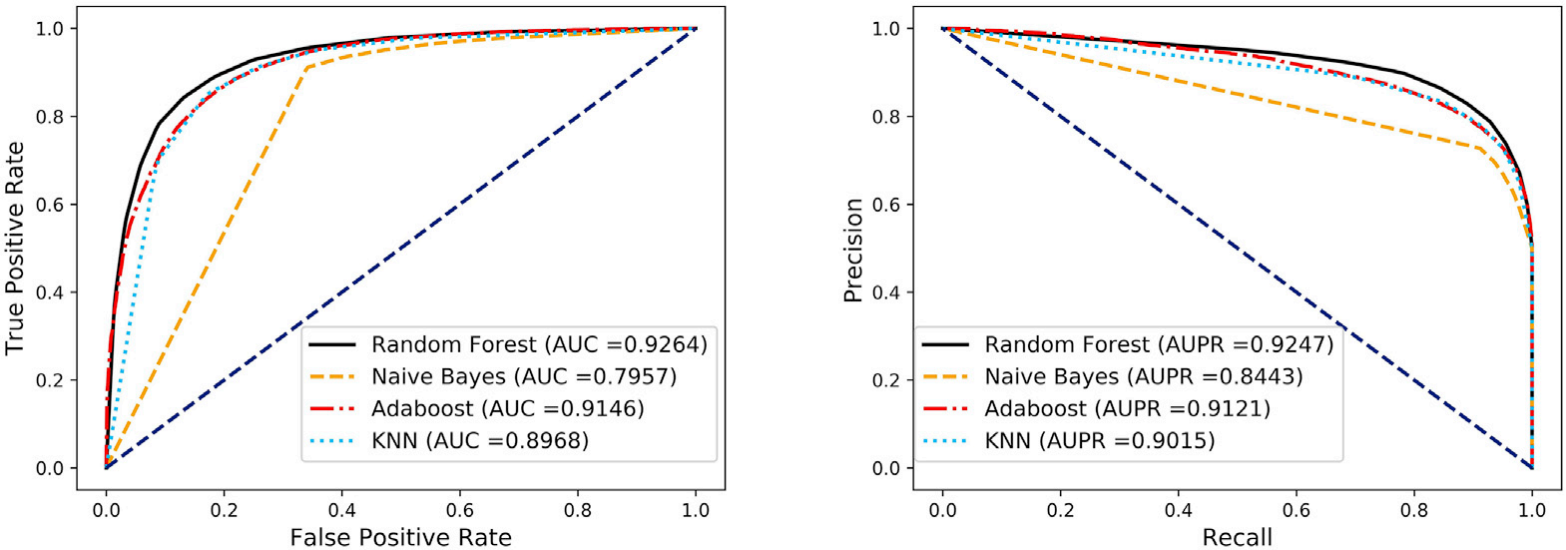
The average ROC and PR curves of DANE-MDA with different classifiers under 5-fold cross-validation

For a specific classification problem, it is crucial to choose a suitable classifier. In this part, we selected four commonly used classifiers for comparison, including Naive Bayes (NB) (Rish, 2001), Adaptive Boosting (AdaBoost) (Margineantu and Dietterich, 1997), K-Nearest Neighbors (KNN) (Denoeux, 2008), and Random Forest (RF) (Liaw and Wiener, 2002), and then used the most suitable classification algorithm to build the prediction model according to the final prediction effect. To make the comparison experiment fair and easy to operate, we kept the experimental environment consistent and performed 5-fold cross-validation for different classifiers with default parameters. Finally, the average results and standard deviations of each classifier under 5-fold cross-validation were recorded in Table 7. Moreover, the ROC and PR curves of the classifier comparison experiment are shown in Figure 10. All the experiments proved that the Random Forest classifier achieved better prediction results and was more suitable for our training model.

**Table 7:** The average results and standard deviations of DANE-MDA with different classifiers under 5-fold cross-validation

Classifier	ACC.(%)	AUC(%)	Sen.(%)	Prec.(%)	Spec.(%)	MCC(%)
KNN	82.69 ± 0.30	89.68 ± 0.39	91.39 ± 0.39	77.85 ± 0.27	74.00 ± 0.35	66.39 ± 0.61
Naive Bayes	78.02 ± 0.44	79.57 ± 0.33	91.77 ± 0.43	71.97 ± 0.35	64.27 ± 0.46	58.28 ± 0.90
AdaBoost	83.56 ± 0.58	91.47 ± 0.22	85.41 ± 0.75	82.36 ± 0.68	81.70 ± 0.83	67.16 ± 1.16
**RandomForest**	**85.59 ± 0.37**	**92.64 ± 0.22**	**84.23 ± 0.77**	**86.60 ± 0.34**	**86.96 ± 0.41**	**71.22 ± 0.72**

### Comparison of previous related works

In the field of potential miRNA-disease association prediction, a lot of excellent computational methods have been developed. To confirm the superiority of our model, we further compared the prediction performance of DANE-MDA based on the HMDD v3.0 with five previous state-of-the-art computational methods, including WBSMDA (Chen et al., 2016), PBMDA (You et al., 2017), HDMP (Xuan et al., 2013), RLSMDA (Chen and Yan, 2014), and DBMDA (Zheng et al., 2020b). WBSMDA predicts the potential associations between miRNAs and diseases by utilizing a model of within and between scores. PBMDA is a path-based prediction method by incorporating multiple similarities of miRNAs and diseases. HDMP is a weighted k-most similar neighbors-based miRNA-disease association prediction method, which is a representative method in this field. RLSMDA is a global, semi-supervised, and regularized least squares-based prediction method. DBMDA utilizes the chaos game representation method based on miRNA sequences and infers global similarity from regional distances to predict miRNA-disease associations. All these methods utilized the known miRNA-disease associations in HMDD v3.0 as the dataset and were verified with the 5-fold cross-validation experiment. Hence, we adopted the average AUC value reported in their article as the evaluation index, as shown in Table 8. Moreover, we also compared the prediction performance of DANE-MDA based on the HMDD v2.0 with the following latest four models, which have been confirmed to achieve excellent prediction accuracy, including TLHNMDA (Chen et al., 2018a), NCMCMDA (Chen et al., 2021), RFMDA (Chen et al., 2018b), and MDHGI (Chen et al., 2018c). Here we also computed the average AUC under the 5-fold cross-validation as the evaluative criterion, and greater AUC means the model shows more accurate prediction performance. Table 9 clearly shows that DANE-MDA achieved better AUC performance under the 5-fold cross-validation based on the HMDD v2.0 dataset. In short, we can clearly observe that DANE-MDA performs better than the current model in potential miRNA and disease association predictions under the 5-fold cross-validation based on both the HMDD v3.0 and v2.0 datasets.

**Table 8:** Comparison of the average AUC value of DANE-MDA and different models based on HMDD v3.0 dataset

Models	Average AUC (%)
DBMDA	91.29
WBSMDA	81.85
PBMDA	91.72
HDMP	83.42
RLSMDA	85.69
**SAE-MDA**	**92.64**

**Table 9:** Comparison of the average AUC value of DANE-MDA and different models based on HMDD v2.0 dataset

Models	Average AUC (%)
TLHNMDA	87.95
NCMCMDA	89.42
RFMDA	88.18
MDHGI	87.94
**SAE-MDA**	**91.13**

### Case studies

In this part, to evaluate the capability of DANE-MDA for predicting potential miRNA-disease associations in practical applications, case studies were conducted on breast neoplasms, colon neoplasms, and lung neoplasms. First, all known and the same number of randomly constructed unknown miRNA-disease associations were constituted as the training samples. Second, the test samples of miRNA-corresponding disease association pairs were, respectively, constituted. It should be noted that the association pairs that already existed in the training samples have been deleted from the test samples. Finally, DANE-MDA was trained based on the training dataset, and then the association probability of unknown miRNA-disease pairs in the test dataset was predicted. On this basis, we listed the top 50 association pairs according to the prediction scores and confirmed them in two other authoritative databases, miR2Disease (Jiang et al., 2008) and dbDEMC (Yang et al., 2010).

Colon neoplasms are the third leading cause of cancer-related deaths in the United States (Siegel et al., 2016). It is a malignant tumor arising from the inner wall of the large intestine (colon) or rectum. The common risk factors for colon neoplasms include colon polyps, family history, age, African American race, and long-standing ulcerative colitis. miRNAs play an essential part in the carcinogenesis and development of colon neoplasms, and their biomarkers have great advantages in the recurrence prediction, diagnosis, and treatment. In this article, DANE-MDA was used to predict the possible miRNAs related to colon neoplasms, and 47 of the top 50 miRNAs with the highest final prediction score were verified as shown in Table 10.

**Table 10:** The top 50 miRNA-colon neoplasm associations predicted by DANE-MDA

Rank	miRNA	Evidence	Rank	miRNA	Evidence
1	hsa-miR-29c-5p	dbDemc	26	hsa-miR-199a-5p	dbDemc
2	hsa-miR-99b-5p	dbDemc	27	hsa-miR-19b-3p	dbDemc
3	hsa-miR-144-5p	dbDemc	28	hsa-miR-497-5p	dbDemc
4	hsa-miR-182-5p	dbDemc	29	hsa-miR-30e-5p	dbDemc
5	hsa-miR-92a-2-5p	dbDemce	30	hsa-miR-27b-5p	dbDemc
6	hsa-miR-338-5p	dbDemc	31	hsa-miR-206	dbDemc
7	hsa-miR-422a	dbDemc; miR2Disease	32	hsa-miR-185-5p	dbDemc
8	hsa-miR-199b-5p	dbDemc	33	hsa-miR-425-5p	dbDemc
9	hsa-miR-378a-5p	dbDemc	34	hsa-miR-135a-5p	dbDemc
10	hsa-miR-373-5p	Unconfirmed	35	hsa-miR-491-5p	dbDemc
11	hsa-miR-451a	dbDemc	36	hsa-miR-340-5p	dbDemc
12	hsa-miR-29b-2-5p	dbDemc	37	hsa-miR-149-5p	dbDemc
13	hsa-miR-214-5p	dbDemc	38	hsa-miR-187-5p	dbDemc
14	hsa-miR-503-5p	dbDemc	39	hsa-miR-129-5p	dbDemc
15	hsa-miR-28-5p	dbDemc	40	hsa-miR-184	dbDemc
16	hsa-miR-146b-5p	dbDemc	41	hsa-miR-95-5p	Unconfirmed
17	hsa-miR-590-5p	dbDemc	42	hsa-miR-7-2-3p -7-2-3p	Unconfirmed
18	hsa-miR-342-5p	dbDemc	43	hsa-miR-7-1-3p	dbDemc
19	hsa-miR-193a-5p	dbDemc	44	hsa-miR-582-5p	dbDemc
20	hsa-miR-421	dbDemc	45	hsa-miR-16-5p	dbDemc
21	hsa-miR-186-5p	dbDemc	46	hsa-miR-10a-5p	dbDemc
22	hsa-miR-26a-5p	dbDemc	47	hsa-miR-181a-2-3p	dbDemc
23	hsa-miR-26b-5p	dbDemc	48	hsa-miR-423-5p	dbDemc
24	hsa-miR-124-5p	dbDemc	49	hsa-miR-181c-5p	dbDemc
25	hsa-miR-122-5p	dbDemc	50	hsa-miR-20b-5p	dbDemc

Breast neoplasms are the most common non-skin malignant tumor in women. In almost all cases it occurs in women, but men can also get breast neoplasms (Bray et al., 2018; Kelsey and Horn-Ross, 1993; Tao et al., 2015). It can begin in different parts of the breast and spread outside the breast through blood and lymph vessels. In addition, more and more studies have shown that miRNAs are a new tool for the prognosis and diagnosis of patients with breast neoplasms. Hence, the prediction of potential breast neoplasms-related miRNAs may identify a novel candidate miRNA for early diagnosis and prevention of breast cancer. In this article, DANE-MDA was used to predict possible miRNAs related to breast neoplasms, and 47 of the top 50 miRNAs with the highest final prediction score were verified as shown in Table 11.

**Table 11:** The top 50 miRNA-breast neoplasm associations predicted by DANE-MDA

Rank	miRNA	Evidence	Rank	miRNA	Evidence
1	hsa-miR-15a-5p	dbDemc	26	hsa-miR-582-5p	dbDemc
2	hsa-miR-181d-5p	dbDemc	27	hsa-miR-1271-5p	dbDemc
3	hsa-miR-99b-5p	dbDemc	28	hsa-miR-1231	dbDemc
4	hsa-miR-500a-5p	dbDemc	29	hsa-miR-589-5p	dbDemc
5	hsa-miR-637	dbDemce	30	hsa-miR-650	dbDemc
6	hsa-miR-454-5p	dbDemc	31	hsa-miR-376a-2-5p	Unconfirmed
7	hsa-miR-646	dbDemc	32	hsa-miR-323b-5p	dbDemc
8	hsa-miR-767-5p	dbDemc	33	hsa-miR-384	dbDemc
9	hsa-miR-28-5p	dbDemc	34	hsa-miR-543	dbDemc
10	hsa-miR-382-5p	dbDemc	35	hsa-miR-302e	dbDemc
11	hsa-miR-508-5p	dbDemc	36	hsa-miR-19b-2-5p	dbDemc
12	hsa-miR-211-5p	dbDemc	37	hsa-miR-337-5p	dbDemc
13	hsa-miR-431-5p	dbDemc	38	hsa-miR-557	dbDemc
14	hsa-miR-532-5p	dbDemc	39	hsa-miR-602	dbDemc
15	hsa-miR-483-5p	dbDemc	40	hsa-miR-154-5p	dbDemc
16	hsa-miR-1297	dbDemc	41	hsa-miR-361-5p	dbDemc
17	hsa-miR-519a-5p	Unconfirmed	42	hsa-miR-4732-5p	dbDemc
18	hsa-miR-501-5p	dbDemc	43	hsa-miR-941	dbDemc
19	hsa-miR-628-5p	dbDemc	44	hsa-miR-362-5p	dbDemc
20	hsa-miR-455-5p	dbDemc	45	hsa-miR-297	dbDemc
21	hsa-miR-601	dbDemc	46	hsa-miR-513c-5p	Unconfirmed
22	hsa-miR-622	dbDemc	47	hsa-miR-571	dbDemc
23	hsa-miR-422a	dbDemc	48	hsa-miR-544a	dbDemc
24	hsa-miR-300	dbDemc	49	hsa-miR-636	dbDemc
25	hsa-miR-325	dbDemc	50	hsa-miR-3651	dbDemc

Lung neoplasms are the leading cause of cancer deaths in men and women. It is usually formed in air passage cells or lung tissue. Factors affecting lung neoplasms mainly include smoking, secondhand smoke, family history of lung cancer, air pollution, HIV infection, etc., among which smoking is the most important risk factor for lung neoplasms (Torre et al., 2016). miRNAs have been determined to play a key role in the treatment and development of lung neoplasms. Compared with normal tissues, the expression level of miRNA in lung cancer cells and the blood of patients with lung cancer are unregulated. Moreover, the phenotype of lung cancer can be changed by regulating miRNA expression both *in vivo* and *in vitro*. In this article, DANE-MDA was used to predict possible miRNAs related to lung neoplasms, and 46 of the top 50 miRNAs with the highest final prediction score were verified as shown in Table 12.

**Table 12:** The top 50 miRNA-lung neoplasm associations predicted by DANE-MDA

Rank	miRNA	Evidence	Rank	miRNA	Evidence
1	hsa-miR-15b-5p	dbDemc	26	hsa-miR-16-2-3p	dbDemc
2	hsa-miR-16-1-3p	dbDemc	27	hsa-miR-425-5p	dbDemc; miR2Disease
3	hsa-miR-518b	dbDemc	28	hsa-miR-484	dbDemc
4	hsa-miR-642a-5p	dbDemc	29	hsa-miR-575	dbDemc
5	hsa-miR-429	dbDemc; miR2Disease	30	hsa-miR-452-5p	dbDemc
6	hsa-miR-106b-5p	dbDemc	31	hsa-miR-590-5p	dbDemc
7	hsa-miR-424-5p	dbDemc	32	hsa-miR-625-5p	dbDemc
8	hsa-miR-28-5p	dbDemc	33	hsa-miR-193b-5p	dbDemc
9	hsa-miR-382-5p	dbDemc	34	hsa-miR-302c-5p	Unconfirmed
10	hsa-miR-409-5p	dbDemc	35	hsa-miR-505-5p	dbDemc
11	hsa-miR-421	dbDemc	36	hsa-miR-181b-5p	dbDemc
12	hsa-miR-532-5p	dbDemc	37	hsa-miR-708-5p	dbDemc
13	hsa-miR-483-5p	dbDemc	38	hsa-miR-1246	dbDemc
14	hsa-miR-128-3p	dbDemc	39	hsa-miR-151a-5p	dbDemc
15	hsa-miR-491-5p	dbDemc	40	hsa-miR-376c-5p	dbDemc
16	hsa-miR-885-5p	dbDemc	41	hsa-miR-370-5p	dbDemc
17	hsa-miR-92b-5p	Unconfirmed	42	hsa-miR-298	dbDemc
18	hsa-miR-509-5p	dbDemc	43	hsa-miR-23b-5p	dbDemc
19	hsa-miR-1307-5p	dbDemc	44	hsa-miR-628-5p	dbDemc
20	hsa-miR-455-5p	dbDemc	45	hsa-miR-539-5p	dbDemc
21	hsa-miR-489-5p	Unconfirmed	46	hsa-miR-711	Unconfirmed
22	hsa-miR-422a	dbDemc	47	hsa-miR-1179	dbDemc
23	hsa-miR-1271-5p	dbDemc	48	hsa-miR-1244	dbDemc
24	hsa-miR-125b-2-3p	dbDemc	49	hsa-miR-339-5p	dbDemc
25	hsa-miR-181d-5p	dbDemc	50	hsa-miR-3613-5p	dbDemc

## Discussion

Recently, an increasing number of researches have demonstrated that miRNAs could fulfill a variety of biological functions, and their abnormal expression or function may cause various human diseases. Thus, the prediction of potential miRNA-disease associations will significantly contribute to the treatment and investigation of complex human diseases. Otherwise, traditional biological experiments are generally laborious and expensive, which leads to a very limited number of experimentally verified miRNA-disease associations. In this study, we propose a computational machine learning-based method (DANE-MDA) that preserves integrated structure and attribute features via deep attributed network embedding and the deep stacked auto-encoder neural network to predict potential miRNA-disease associations. Specifically, the DANE-MDA framework is composed of four steps. First, the network structure and attribute feature of diseases and miRNAs is respectively calculated. Second, the interactions between network structure and attribute information of miRNAs and diseases from diverse degrees of proximity are captured by utilizing a personalized random walk-based method. Third, we fuse the diverse degrees of proximity to build an enhanced matrix representation to preserve both the attribute information and the local and global network structure features and then utilized the deep stacked auto-encoder to learn the complex nonlinear information of the enhanced matrix to represent miRNAs and diseases. Finally, the potential miRNA-disease association prediction approach is built based on the Random Forest classifier. The prediction results under 5-fold cross-validation confirmed the excellent capability of DANE-MDA. Moreover, we also discussed the influence of parameters and classifiers on the final prediction results. Last, the case studies performed on three complex human diseases once again demonstrated the good property of DANE-MDA in practical applications.

### Limitations of the study

There are still some limitations in the current method that should to be addressed. First, in terms of attribute feature extraction, we hope to make full use of various information in the future, such as miRNA functional similarity and Gaussian interaction profile kernel similarity, rather than just the sequence and semantic information of miRNAs and diseases. Second, in terms of advanced feature extraction and avoiding the curse of dimensionality, we hope to compare deep stacked auto-encoder with other deep neural network learning algorithms in the future to achieve better performance. Third, DANE-MDA is a computational machine learning-based prediction model. Hence, a suitable machine learning classifier is essential for our predictive model. We hope to consider other new classifiers to improve prediction ability in the future instead of using the old model such as random forest.

### Resource availability

#### Lead contact

Further information and requests for resources should be directed to and will be fulfilled by the lead contact, Zhu-Hong You (zhuhongyou@ms.xjb.ac.cn).

#### Materials availability

In this study, the known miRNA-disease association dataset was first selected from the Human MicroRNA Disease Database (HMDD) v3.0 (Huang et al., 2019), which is a public online database that contains 32,281 experimentally affirmed miRNA-disease associations from 17,412 papers, containing 850 diseases and 1,102 miRNAs. On this basis, we conducted data preprocessing to eliminate duplicate associations and delete the associations related to certain miRNAs considered unreliable by the public database miRBase (Griffiths-Jones et al., 2006). Finally, 16,427 miRNA-disease associations containing 850 diseases and 901 miRNAs were acquired as the positive samples. Additionally, the Human MicroRNA Disease Database (HMDD) v2.0 dataset was downloaded from the http://www.cuilab.cn/static/hmdd3/data/hmdd2.zip, including 5,430 experimentally verified human miRNA-diseases associations about 383 diseases and 495 miRNAs. For the negative samples, we adopted most previous methods that utilize random selection to generate them with the same number as positive samples (Ben-Hur and Noble, 2005).

#### Data and code availability

The datasets generated and/or analyzed during this study are available under open licenses in the data repository, https://github.com/jiboya123/DANE-MDA.

## Methods

All methods can be found in the accompanying Transparent Methods supplemental file.

## Supplemental information

Document S1. Transparent methods and figures S1–S4
