# Supplementary material for: DANE-MDA: Predicting microRNA-disease associations via deep attributed network embedding
Source: iScience. 2021 Apr 20;24(6):102455. doi: 10.1016/j.isci.2021.102455 (PMC8141887; doi:10.1016/j.isci.2021.102455)
Supplement: Document S1. Transparent methods and figures S1–S4 [file mmc1.pdf]

iScience, Volume *24*

Supplemental information

DANE-MDA: Predicting microRNA-disease associations via deep attributed network embedding

Bo-Ya Ji, Zhu-Hong You, Yi Wang, Zheng-Wei Li, and Leon Wong

Supplemental Figures

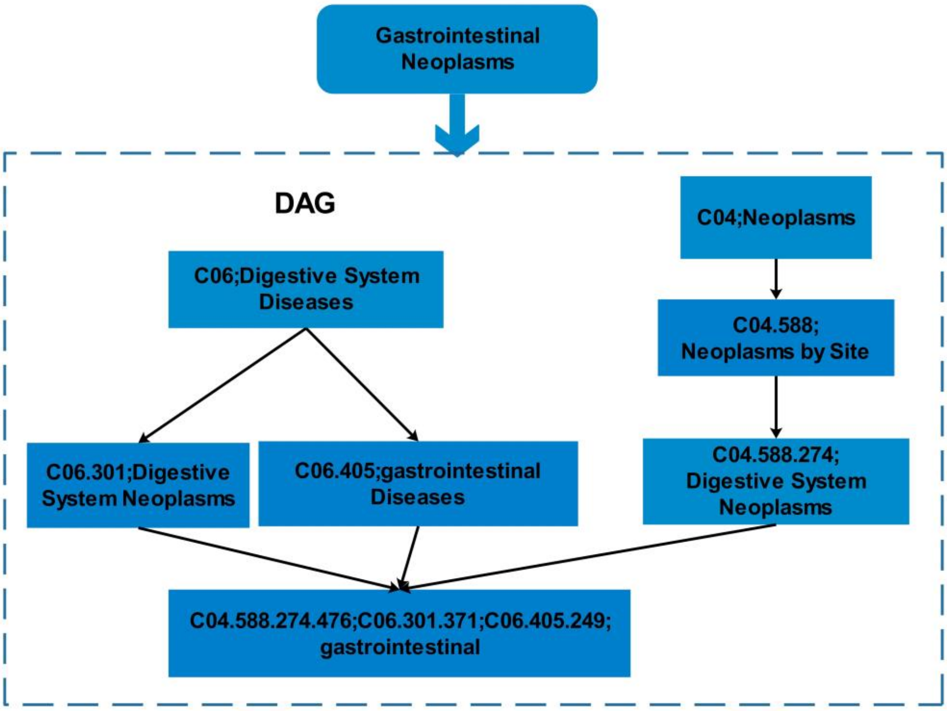

Figure S1. The directed acyclic graph (DAG) of gastrointestinal neoplasms. Related to Figure 1.

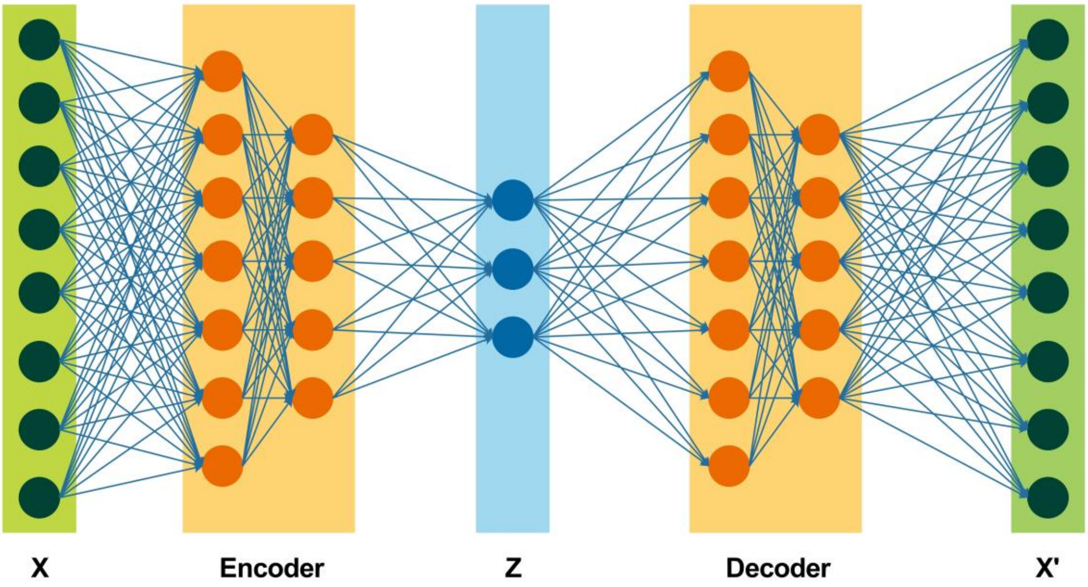

Figure S2. The simplified schematic diagram of deep stacked auto-encoder neural network. Related to Figure 1.

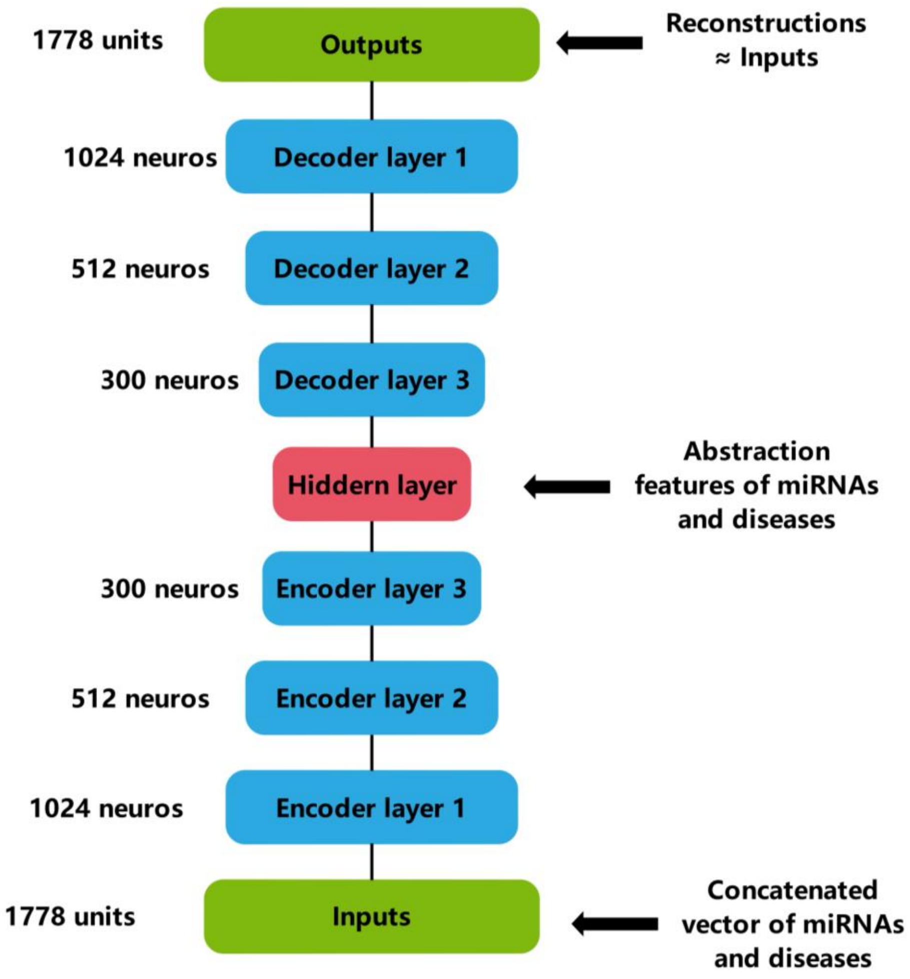

Figure S3. The architecture of our deep stacked auto-encoder neural network model. Related to Figure 1.

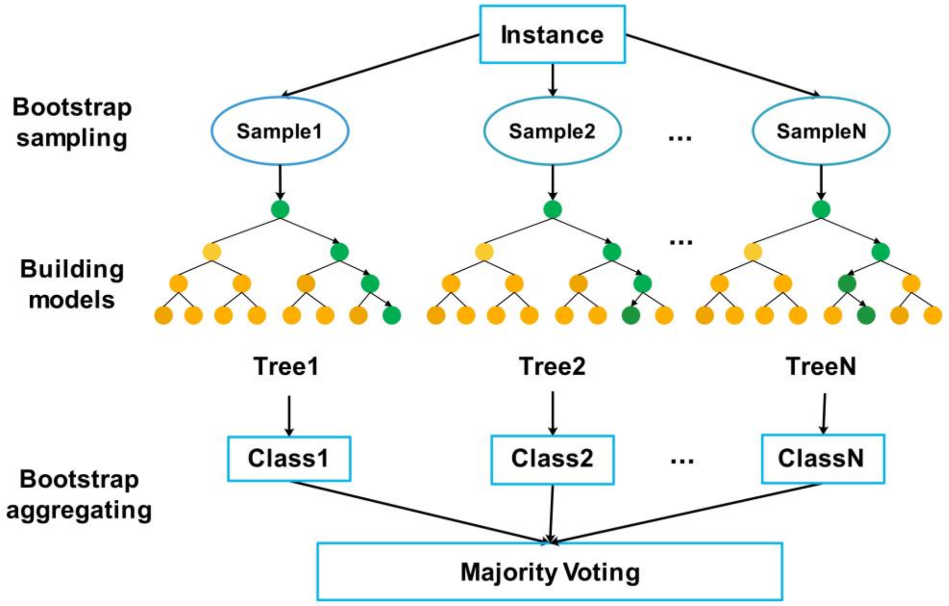

Figure S4. The simplified flowchart of the Random Forest classifier. Related to Figure 1.

Transparent Methods

miRNA sequence similarity information

In this study, the attribute feature of miRNAs was represented by the sequence similarity information. Generally, miRNA sequences are usually denoted by simplified letters of four nitrogenous bases: uracil (U), cytosine (C), guanine (G), and adenine (A). We downloaded the miRNA sequence information from the public miRBase database (Griffiths-Jones et al., 2006) and then utilized the 3-mer method to obtain the numerical statistical features of miRNA sequences. Specifically, we first set up a sliding window with a window size of 3 and a sliding distance of 1, to split the miRNA sequence into multiple 3-monomeric units (3-mers). Second, the occurrence number of each 3-mer is divided by the corresponding miRNA sequence length to obtain its occurrence frequency, and the occurrence frequency of non-occurring 3-mers is set to 0. Finally, each miRNA sequence is converted into a 64-dimensional vector based on the 64 kinds of 3-mer combinations. On this basis, we continue to use the most common similarity measurement method Euclidean distance to calculate the miRNA sequence similarity (MSS), defined as follows:

$$\mathrm{Sim}(\mathrm{M},\ \mathrm{M}') = \sqrt{\sum_{i=1}^{n}(M_i - M'_i)^2} \quad (1)$$

$$\mathrm{MSS}(\mathrm{M}) = \left(\mathrm{Sim}(M, M'_1), \mathrm{Sim}(M, M'_2), \dots, \mathrm{Sim}(M, M'_m)\right) \quad (2)$$

where M and M' represent the numerical statistical feature vectors of two miRNA sequences, n represents the vector length, and m means the number of miRNAs.

Disease semantic similarity

In this study, the disease semantic similarity was used to represent the attribute feature of diseases. The Medical Subject Heading (MeSH) descriptors of diseases provide a strict disease classification system, which can be obtained from the U.S. National Library of Medicine (https://www.nlm.nih.gov/) (Lipscomb, 2000). MeSH descriptors

are divided into 16 categories: category A is anatomical terms, category B is organisms, category C used in this study is disease terms, and so on. On this basis, the relationship among various diseases can be represented as a directed acyclic graph (DAG), where the nodes represent the MeSH descriptors of the diseases, and the directed edges point from more general items (parent nodes) to more specific ones (child nodes). Besides, there are one or more tree numbers of each MeSH descriptor to indicate its position in the DAGs. The child node's tree number is its parent node's tree number appended by its information. Figure S1 shows an example of the DAG for gastrointestinal neoplasms. For instance, disease A can be defined as DAG(A) = (D(A), E(A)), in which D(A) is meant as A and its ancestor nodes, and E(A) is meant as all the direct edges. On this basis, the semantic contribution of disease term *t* in DAG(A) to disease A is defined as follows:

$$\begin{cases} D_A(t) = 1 & if\ t = A \\ D_A(t) = max\{\Delta * D_A(t') | t' \in children\ of\ t\} & if\ t \neq A \end{cases} \quad (3)$$

where Δ is the semantic contribution attenuation factor, which means that its semantic contribution to disease A will decrease as the distance between item *t* and disease A increases. Disease A is at the bottom of the DAG, so we defined its contribution value as 1. According to the above formula, the contribution of items at different levels to the semantic value of disease A can be differentiated. Finally, the semantic value of disease A is achieved by summarizing all the contributions from itself and its ancestor diseases, as shown below:

$$\mathrm{DV(A)} = \sum_{t \in D(A)} D_A(t) \quad (4)$$

Hence, the disease semantic similarity (DSS) between diseases d_i and d_j is acquired based on the nodes shared by the two disease DAGs as follows:

$$\mathrm{DSS(d_i, d_j)} = \frac{\sum_{t \in D(d_i) \cap D(d_j)} (D_{d_i}(t) + D_{d_j}(t))}{DV(d_i) + DV(d_j)} \quad (5)$$

Network structure feature of miRNAs and diseases

In this study, the local network structure feature of miRNAs and diseases was represented by the probability of direct transitions between each miRNA-disease association pair. First, we generated an adjacency matrix *R* based on the constructed attributed miRNA-disease association network. The row and column number of *R* is 901 and 850, representing the number of miRNAs and diseases. The element R_{ij} in the matrix represents the relationship between miRNA m_i and disease d_j. If there is an association between m_i and d_j, the R_{ij} is equal to 1, otherwise, equal to 0. Second, we normalized the adjacency matrix *R* by row to generate the network structure feature matrix *S*, which shows the connection probability between miRNAs and diseases within one step, given by:

$$S_{ij} = \frac{R_{ij}}{\sum_{k \in N} R_{ik}} \tag{6}$$

where *N* is the column number of matrix *R*, and S_{ij} is the associated probability of miRNA m_i and disease d_j. Thus, the structural feature matrix *S* should satisfy the following constraints:

$$0 \leqslant S_{ij} \leqslant 1 \tag{7}$$

$$\forall i \in [1, 2, \cdots, N], \quad \sum_{k=1}^{N} S_{ij} = 1 \tag{8}$$

Construct the attribute and structure matrix representation

The attribute matrix representation *A* for the attributed network is formed by combining the miRNA sequence similarity matrix *RM* and disease semantic similarity matrix *RD*. Moreover, since there is no attribute relationship between miRNAs and diseases, we set this part as the 0 matrices. The final attribute matrix representation is defined as follows:

$$A = \begin{vmatrix} RM & 0 \\ 0 & RD \end{vmatrix} \tag{9}$$

The network structure matrix *S* is composed of the probability of direct transition between each miRNA-disease association pair. Similarly, since there is no structural

relationship between miRNAs and diseases themselves, we also set this part as the 0 matrices. The final structure matrix representation is defined as follows:

$$\mathrm{S} = \begin{vmatrix} 0 & S \\ S^T & 0 \end{vmatrix} \quad (10)$$

where S^T is represented the transposed matrix of network structure matrix S. The number of rows and columns of the network structure matrix are both the sum of the number of miRNAs and diseases.

Step-based proximity calculation

For the purpose of catching the interactions between the attribute and network structure feature from diverse degrees of proximity, the graph-based random walk idea was borrowed to construct a step-based proximity matrix P^t at each step *t*. The first-degree proximity matrix P^1 is meant as the linear combination of the attribute feature matrix *A* and the network structure feature matrix *S*, in which only the first-order proximity between miRNA m_i and disease d_j in the network structure is considered, given by:

$$P^1_{(i,j)} = \alpha S(\mathrm{i},\mathrm{j}) + (1-\alpha)\mathrm{A}(\mathrm{i},\mathrm{j}) \quad (11)$$

where $\alpha \in (0, 1)$ is the weight coefficient, which means the random walk preference ratio between attribute and structure feature matrix.

Furthermore, in order to catch the higher-degree structure proximity, we defined the (*t+1*)-th step-based proximity P^{t+1} as:

$$P^{t+1} = \alpha P^t \mathrm{S} + (1-\alpha)A \quad (12)$$

Specifically, the (*t+1*)-th step structure proximity was obtained by multiplying the *t*-th step proximity matrix P^t by the structure matrix S, and since the attribute features are static in network structure changes, the attribute proximity is always A. In this way, we obtained both the attribute features and the local and global structure features of the network from different degrees of proximity with the proximity matrix sequences: $P^1, P^2, \ldots, P^t$.

Diverse degrees of proximity fusion

In this part, to preserve both the attribute features, as well as the local and global network structure feature of miRNAs and diseases, an enhanced matrix Q is constructed by fusing the diverse degrees of proximity: $P^1, P^2, \ldots, P^t$. Generally, it is a common fusion strategy to average the sum of all matrices. But intuitively, the closer (the smaller the degree) the connections between miRNAs and diseases, the closer the relationship between them. In other words, the low-order proximity nodes have a greater influence than high-order proximity ones. Hence, a weight function that decreases monotonously with the increase of step *t* is defined as:

$$Q = \sum_{t=1}^{T} f(t) * P^t \tag{13}$$

where *f(t)* represents a decreasing function, and in this study, an exponential function modified by the parameter $\beta \in (0, 1)$ is used as the weighting strategy as shown below:

$$f(t) = \beta^t \tag{14}$$

Deep stacked auto-encoder neural network

In order to improve feature quality and reduce noise, we further learned the nonlinear and complex low-dimensional features in the fusion matrix Q. The deep stacked auto-encoder neural network (SAE) (Rumelhart et al., 1986) is utilized to obtain the embedding features of miRNAs and diseases. Specifically, SAE is a category of unsupervised learning for data compression, and the simplified SAE is a three-layer neural network model, including a data input layer, a hidden layer, and an output reconstruction layer. The encoding process is used to map the input data from the input layer to the hidden layer, and the decoding process is used to map the hidden data from the hidden layer to the output layer to reconstruct the input data. The schematic diagram of the simplified deep stacked auto-encoder is shown in Figure S2. Given the input data:

$$x = [x_1, x_2, \dots, x_{d(x)}]^T \in R^{d(x)} \quad (15)$$

where *d(x)* means the dimension of the input data, and then the *x* is projected by the encoder from the input layer to the hidden layer data *z* with the mapping function *f* :

$$z = [z_1, z_2, \dots, z_{d(z)}]^T \in R^{d(z)} \quad (16)$$

where *d(z)* means the dimension of the hidden layer data, and *f(x)* function is expressed as:

$$z = f(x) = s_f(Wx + b) \quad (17)$$

where $W \in R^{d(x)*d(z)}$ is the weight matrix, $b \in R^{d(x)}$ is the deviation vector. The activation function s_f of the encoder can be a sigmoid function, a tanh function, or a rectified linear unit function (ReLu function).

In the decoder, the hidden layer representation *z* is mapped to the output layer $x' \in R^{d(x')}$ through the mapping function f', where the function is as follows:

$$x' = f'(z) = s_{f'}(W'z + b') \quad (18)$$

where $W' \in R^{d(x')*d(z)}$ is the weight matrix, $b' \in R^{d(x')}$ is the deviation vector. Similarly, the activation function $s_{f'}$ of the decoder can also be a sigmoid, tanh, or ReLu function. Thus, the parameter set of SAE is:

$$\theta = \{W, W', b, b'\} \quad (19)$$

To obtain the optimal model parameters, the loss function is reconstructed by computing the mean square reconstruction error to minimize:

$$J(W, W', b, b') = \frac{\sum_{i=1}^{N} \|x_i' - x_i\|^2}{2N} \quad (20)$$

where *N* is the total number of training samples. Figure S3 shows the architecture of our stacked auto-encoder model. Specifically, the input layer of the model is a concatenated vector of diseases and miRNAs. The encoder part contains a total of 3

layers, each containing 1024, 512, and 300 neurons. The decoder part has the reverse architecture of the encoder, each containing 300, 512, and 1024 neurons. Moreover, we set nonlinear activation functions to ReLU, the loss function to the mean squared error (MSE), which is minimized using Adam, the epochs to 100, and the batch size to 128.

Random Forest classifier

Generally, determining whether there is an association between miRNAs and diseases is regarded as a binary classification problem. In this study, the Random Forest (RF) classifier (Liaw and Wiener, 2002) is chosen for training the deep attributed network embedding features of miRNAs and diseases and predicting potential associations between them. In particular, Random Forest is a significant bagging-based ensemble learning method and has a lot of advantages, such as high accuracy rate, not easy to overfit, and good anti-noise ability, which could be utilized for regression, classification, and other problems. Its construction process is roughly as follows: (1) Generate *N* samples from the instance by utilizing the bootstrap sampling method. (2) Establish *N* decision tree models based on *N* training samples. For a single decision tree model, the best feature is used for each split according to the Gini index/information gain ratio/ information gain. (3) Use the majority voting mechanism to determine the final prediction result. Figure S4 shows the simplified flowchart of the Random Forest classifier.

Supplemental References

Griffiths-Jones, S., Grocock, R.J., Van Dongen, S., Bateman, A., and Enright, A.J. (2006). miRBase: microRNA sequences, targets and gene nomenclature. Nucleic acids research *34*, D140-D144.

Liaw, A., and Wiener, M. (2002). Classification and regression by randomForest. R news *2*, 18-22.

Lipscomb, C.E. (2000). Medical subject headings (MeSH). Bulletin of the Medical Library Association *88*, 265.

Rumelhart, D.E., Hinton, G.E., and Williams, R.J. (1986). Learning representations by back-propagating errors. nature *323*, 533-536.
